# Antibacterial Efficacy of Iron-Oxide Nanoparticles against Biofilms on Different Biomaterial Surfaces

**DOI:** 10.1155/2014/716080

**Published:** 2014-09-23

**Authors:** Monica Thukkaram, Soundarya Sitaram, Sathish kumar Kannaiyan, Guruprakash Subbiahdoss

**Affiliations:** ^1^Department of Biomedical Engineering, SSN College of Engineering, Old Mahabalipuram Road, Kalavakkam, Tamil Nadu 603110, India; ^2^Department of Chemical Engineering, SSN College of Engineering, Old Mahabalipuram Road, Kalavakkam, Tamil Nadu 603110, India

## Abstract

Biofilm growth on the implant surface is the number one cause of the failure of the implants. Biofilms on implant surfaces are hard to eliminate by antibiotics due to the protection offered by the exopolymeric substances that embed the organisms in a matrix, impenetrable for most antibiotics and immune cells. Application of metals in nanoscale is considered to resolve biofilm formation. Here we studied the effect of iron-oxide nanoparticles over biofilm formation on different biomaterial surfaces and pluronic coated surfaces. Bacterial adhesion for 30 min showed significant reduction in bacterial adhesion on pluronic coated surfaces compared to other surfaces. Subsequently, bacteria were allowed to grow for 24 h in the presence of different concentrations of iron-oxide nanoparticles. A significant reduction in biofilm growth was observed in the presence of the highest concentration of iron-oxide nanoparticles on pluronic coated surfaces compared to other surfaces. Therefore, combination of polymer brush coating and iron-oxide nanoparticles could show a significant reduction in biofilm formation.

## 1. Introduction

Biofilm growth on the surface of biomaterial implants is generally recognized as a cause of biomaterial-associated infection (BAI). These infections impose serious complications associated with the use of biomaterial implants. Regardless of the high sterile conditions and improved techniques in the operating theatre, both perioperative and postoperative contamination by microorganisms suspended in the air and from the skin flora continue to be the most common pathway for the contamination of biomaterial implants and medical devices [1, 2]. Microorganisms get adhered to the biomaterial surfaces and grow to form biofilms. The biofilm mode of growth protects the organisms against the host defense system and antibiotics [3]. Therefore complete removal of an infected implant or device is often the final result of BAI.

BAI starts with the initial adhesion of microorganisms and then subsequently grows to form a biofilm. Bacterial adhesion on surfaces is influenced by physicochemical properties of the surface [4]. Surface wettability is one of the important properties influencing bacterial interactions with biomaterials. Gottenbos et al. [5] showed that bacterial adhesion was on materials with different wettabilities. A hydrophilic polymer brush coating is included, since these have been shown to discourage microbial adhesion [6]. Several attempts have been made to develop nonadhesive coatings [7], such as polymer brush coatings, in order to prevent bacterial adhesion and subsequent biofilm growth [8, 9]. Polymer brushes are end tethered polymer chains, having high density of chains per unit surface area due to which it stretches away from a surface into the adjacent solution [10]. Polyethylene oxide (PEO) brush coating forms a highly hydrated layer of chains that is compressed upon bacterial approach, leading to a repulsive osmotic force and weak repulsive forces and reduced mobility of the polymer chains. This creates a steric barrier which discourages close contact and suppresses adhesion [8]. Though most types of brush coatings show significant reductions in microbial adhesion [11–13], bacteria adhere more weakly to the surface [14], being capable of growing into a mature biofilm. Moreover these brush coatings only prevent adhesion and are incapable of killing the bacteria present [15].

Nanoparticles are less than 100 nm in diameter and as a result properties such as surface area, chemical reactivity, and biological activity alter dramatically. The antibacterial efficacy of metal nanoparticles has been suggested to be due to their high surface-to-volume ratio rather than to the sole effect of metal-ion release [16]. A high surface-to-volume ratio is generally accompanied by increased production of reactive oxygen species, including free radicals [17, 18]. These characteristics allow nanoparticles to interact closely with microbial membranes, damaging their structure and inactivate bacteria. Metal oxide nanoparticles are of particular interest as antibacterial agents, as they can be prepared with extremely high surface areas and unusual crystalline morphologies with a high number of edges and corners and other potentially reactive sites [19]. Iron-oxide nanoparticles are a special class of metal oxide nanoparticles with unique magnetic properties and superior biocompatibility. Therefore, the aim of the study was to evaluate the effect of iron-oxide nanoparticles over biofilm formation on different biomaterial and polymer brush coated surfaces. The antimicrobial activity of different concentrations of iron-oxide nanoparticles was assessed.

## 2. Materials and Methods

### 2.1. Biomaterials Surfaces

Poly(methyl methacrylate) (PMMA) (Industrial Insulation, Chennai, India), polystyrene (PS) (Industrial Insulation, Chennai, India), tissue culture polystyrene well plates (TCPS) (NEST Biotech Co. Ltd., China), glass slide (GS, control), and surfaces (PMMA and TCPS) coated with a hydrophilic polyethylene oxide (PEO) layer were used. All samples except hydrophilic PEO coating and TCPS were rinsed thoroughly with ethanol (Jiangsu Huaxi International trade Co. Ltd., China) and washed with sterile water before use.

Hydrophilic PEO-coated surface (polymer brush coating) was prepared by first cleaning the surfaces in sterile water, ethanol, and water again and finally washing with sterile water. Surfaces were made hydrophobic by application of dimethyldichlorosilane coating. Exposure to a solution of 1 g/L pluronic F-68 solution (HIMEDIA Laboratories Pvt. Ltd., Mumbai, India) in phosphate-buffered saline (PBS: 10 mM potassium phosphate, 0.15 M NaCl, pH 7.0) for 20 min created a hydrophilic polymer brush coating over the surface.

### 2.2. Biomaterial Surface Characterization

The wettability of the surfaces was determined by water contact angle measurements at room temperature with an image analyzing system, using sessile drop technique. Each value was obtained by averaging five droplets on one sample.

### 2.3. Synthesis of Iron-Oxide Nanoparticles

4 mL of ferrous chloride and 1 mL of ferric chloride were added to a flask. Sodium hydroxide was added drop by drop and stirred continuously. Initially formed brown precipitate with time should be changed into a black precipitate, indicating the formation of iron-oxide nanoparticles. The size of the synthesized particles was determined using transmission electron microscopy (TEM). The optical measurement of the nanoparticles was studied by UV-visible spectrophotometer (UNICO) over the spectral range of 200–1000 nm.

### 2.4. Bacterial Growth Conditions and Harvesting

*Staphylococcus aureus*,* Escherichia coli*, and* Pseudomonas aeruginosa* were used for this study. Bacterial strains used in this study were obtained from the culture collection of the Centre for Drug Discovery and Development, Sathyabama University, Chennai, India. Bacteria were first grown aerobically overnight at 37°C on blood agar from a frozen stock. The plate was kept at 4°C. For each experiment, one colony was inoculated in 10 mL of tryptone soy broth (TSB; Hi media, Mumbai, India) and cultured for 16 h. Bacteria were harvested by centrifugation at 3000 rpm for 5 min. Bacteria are then suspended in TSB to a concentration of 10^7^ bacteria/mL.

### 2.5. Bacterial Adhesion on Different Surfaces

Bacterial adhesion was performed on six different surfaces (GS, PS, PMMA, polymer brush coated PMMA, TCPS, and polymer brush coated TCPS). Samples were placed in the tissue culture polystyrene well plates. Each well was filled with 1 mL of bacterial suspension and allowed to adhere and grow aerobically at 37°C for 30 min. Bacterial adhesion on GS was considered as control. Subsequently, wells were washed with sterile phosphate buffer saline (10 mM potassium phosphate, 0.15 M NaCl, pH 7.0) to remove unbound bacteria and images were taken using phase contrast microscopy and the number of adherent bacteria per cm^2^ was determined using ImageJ software. Experiments were performed in triplicate with separately cultured bacteria.

### 2.6. Antibacterial Activity of Iron-Oxide Nanoparticles

Freshly prepared nutrient agar plates were used. Bacterial cultures were inoculated to the agar plates and incubated at 37°C for 30 min. Holes of 6 mm diameter were punched into the nutrient agar plates. Holes were filled with 100 *μ*L of iron-oxide nanoparticles (0.01 mg/mL, 0.05 mg/mL, 0.10 mg/mL, and 0.15 mg/mL) and incubated at 37°C for 24 h. The antibacterial activity was assessed by measuring the zone of inhibition.

### 2.7. Effect of Iron-Oxide Nanoparticles over Biofilm Growth on Polymer Brush Coated Surface

In this study, TCPS and polymer brush coated TCPS were compared. 1 mL of bacterial suspension was added to each well and allowed to adhere and grow aerobically at 37°C for 30 min. Then, iron-oxide nanoparticles were introduced in different concentrations (0.01 mg/mL, 0.05 mg/mL, 0.10 mg/mL, and 0.15 mg/mL). Thereafter, biofilms were allowed to grow for 24 h. Subsequently, wells were washed with sterile water to remove unbound bacteria and biofilm development was assessed by measuring the optical density using spectrophotometer. To this end, 500 *μ*L of 0.1% crystal violet staining was added to each well. Plates were incubated for 5 min. Then, crystal violet was removed. The wells were washed with sterile water and 33% acetic acid was added to each well. The optical density (absorbance at 590 nm) was measured using spectrophotometer [20]. Experiments were performed in triplicate with separately cultured bacteria.

### 2.8. Statistical Analysis

Experiments were performed in triplicate. Data are represented as a mean with standard deviation. For statistical analysis ANOVA was performed followed by a Tukey's HSD post hoc test and a *P* value <0.05 was considered to be significant.

## 3. Results 

### 3.1. Biomaterial Surface Wettability

**Figure 1:**
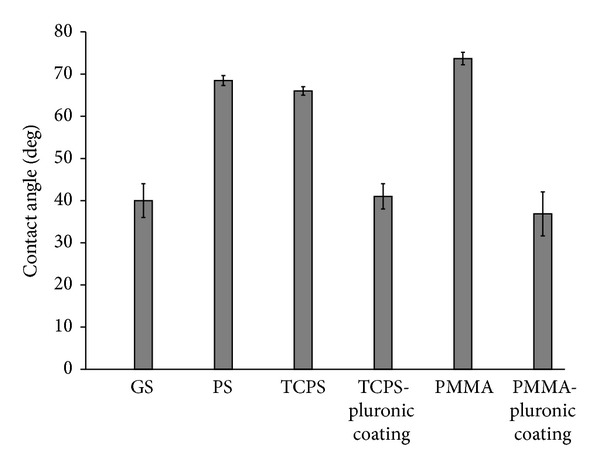
Water contact angle of biomaterial surfaces (GS: glass slide, PS: polystyrene, TCPS: tissue culture polystyrene, and PMMA: poly(methyl methacrylate)) and pluronic coated surfaces.

The water contact angles of biomaterial and polymer brush coated surfaces are shown in Figure 1. The biomaterial surfaces extend over a wettability range from 52° to 73°. The polymer brush coating on PMMA and TCPS has an average wettability of 36° [21] and 41°, respectively.

### 3.2. Iron-Oxide Nanoparticles Characterization

**Figure 2:**
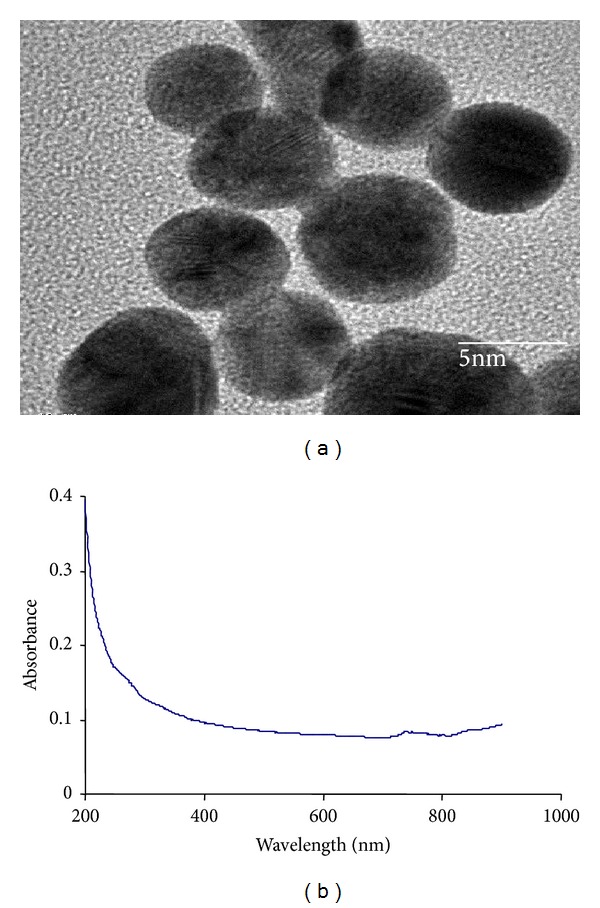
(a) Transmission electron micrograph of iron-oxide nanoparticles. Bar denotes 5 nm. (b) UV-visible spectrum of iron-oxide nanoparticles [23].

The TEM images of synthesized iron-oxide nanoparticles are shown in Figure 2(a). The nanoparticles were measured to be less than 10 nm. The UV-visible spectrum of iron-oxide nanoparticles was shown in Figure 2(b) where the absorbance of nanoparticles steadily decreases with time which confirms the formation of oleic acid coated iron-oxide nanoparticles.

### 3.3. Bacterial Adhesion to Surfaces

**Figure 3:**
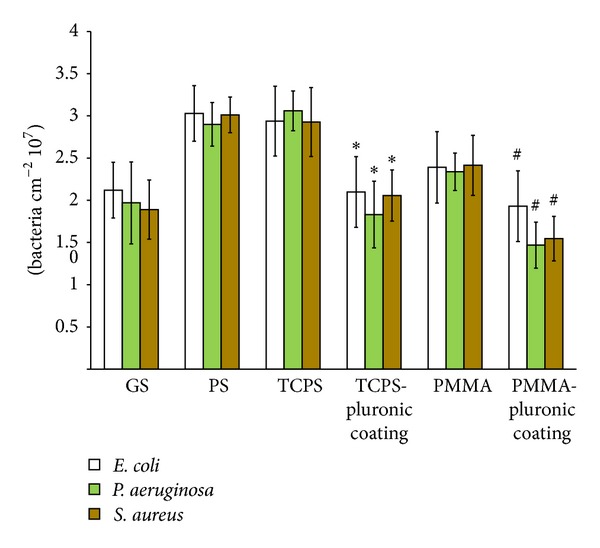
Number of adherent bacteria after 30 min on different biomaterial surfaces (GS: glass slide, PS: polystyrene, TCPS: tissue culture polystyrene, and PMMA: poly(methyl methacrylate)) and pluronic coated surfaces. ∗Significant difference at *P* < 0.05 compared to TCPS surfaces. # denotes significant difference at *P* < 0.05 compared to PMMA surfaces.

Initial adhesion of bacteria after 30 min of incubation was significantly (*P* < 0.05) reduced on polymer brush coated surfaces compared to bare surfaces (Figure 3). This trend holds good for all the three bacteria (*Staphylococcus aureus*,* Escherichia coli*, and* Pseudomonas aeruginosa*) on both PMMA and TCPS surfaces. No significant difference was observed on bare TCPS compared to PS surfaces.

### 3.4. Antibacterial Efficacy of Iron-Oxide Nanoparticles

The antibacterial activity of iron-oxide nanoparticles is shown in Table 1. The zone of inhibition of iron-oxide nanoparticles was directly proportional to the increase in concentration of iron-oxide nanoparticles (Table 1). At 0.15 mg/mL of iron-oxide nanoparticles, the highest inhibition (29 mm) was observed in* S. aureus* compared to* E. coli* and* P. aeruginosa*.

**Figure 4:**
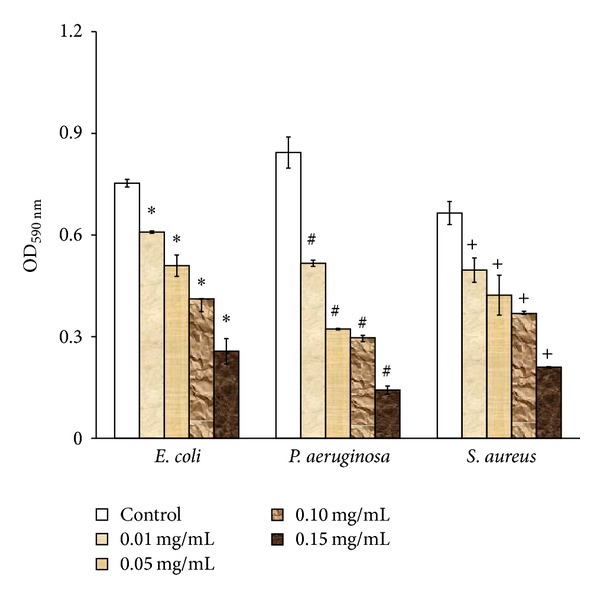
Optical density measurements of 24 h biofilm growth on pluronic coated TCPS surface in the presence of different concentrations (0.01 mg/mL, 0.05 mg/mL, 0.10 mg/mL, and 0.15 mg/mL) of iron-oxide nanoparticles. ∗Significant difference at *P* < 0.05 compared to control (absence of iron-oxide nanoparticles). # denotes significant difference at *P* < 0.05 compared to control (absence of iron-oxide nanoparticles) and + denotes significant difference at *P* < 0.05 compared to control (absence of iron-oxide nanoparticles).

Influence of iron-oxide nanoparticles at different concentrations against biofilm growth on polymer brush coated surface was shown in Figure 4. Significant reduction (*P* < 0.05) in biofilm growth on all the three bacteria was observed in the presence of iron-oxide nanoparticles compared to control (absence of iron-oxide nanoparticles). The highest reduction (*P* < 0.05) was observed in the presence of iron-oxide nanoparticles at 0.15 mg/mL compared to other concentrations (0.01 mg/mL, 0.05 mg/mL, and 0.1 mg/mL) and control.

## 4. Discussion

This paper presents the experimental study on the bacterial adhesion and biofilm growth on various biomaterials including polymer brush coated surfaces and the strategy of using iron-oxide nanoparticles in eradication of biofilms. Biofilm growth on biomaterials is generally the cause of BAI.* Staphylococcus aureus*,* Escherichia coli*, and* Pseudomonas aeruginosa* are the frequently isolated pathogens from infections related to biomaterials implant surfaces [22]. Therefore, these pathogens were considered in our experiments.

Amongst other material properties, surface wettability plays a major role in bacterial adhesion to biomaterials. Wettability of biomaterial surfaces has been related to bacterial adhesion and biofilm growth [16]. Studies showed that staphylococci adhesion to different biomaterials showed no differences irrespective of differences in wettability [5], whereas in our study a significant reduction in bacterial adhesion after 30 min was observed in GS compared to other surfaces (PMMA, TCPS, and PS). And polymer brush coated PMMA and TCPS surfaces showed significant reduction (*P* < 0.05) in bacterial adhesion (*S. aureus*,* E. coli*, and* P. aeruginosa*) compared to bare PMMA and TCPS surfaces. Similarly, Nejadnik et al. [6] showed that the polymer brush coatings reduced adhesion of staphylococci considerably but the few adhered bacteria still formed a biofilm when allowed to grow.

Metals have been used as antibacterial agent for centuries [19] and their efficacy has been surpassed by the use of modern antibiotics. Use of metals in nanoparticulated form is considered to resolve bacterial infections. Taylor and Webster [23] showed that iron-oxide nanoparticles in a concentration range of 0.01 to 2 mg/mL were able to kill up to 25% of* S. epidermidis* in a 48 h old biofilm. And, similar results were observed in our previous and current studies with iron-oxide nanoparticles on* S. aureus* biofilms [24]. In contrast, Haney et al. [25] showed an increase in* P. aeruginosa* biofilm biomass in the presence of 0.2 mg/mL of superparamagnetic iron-oxide nanoparticles.

In this study, influence of iron-oxide nanoparticles on biofilms formed on polymer brush coated biomaterial surface was evaluated. The study of combined effects of polymer brush coating and iron-oxide nanoparticles on biofilms is novel. A significant reduction (*P* < 0.05) in biofilm growth on all the three bacteria was observed in the presence of iron-oxide nanoparticles compared to control (absence of iron-oxide nanoparticles). The highest reduction (*P* < 0.05) was observed in the presence of iron-oxide nanoparticles at 0.15 mg/mL compared to other concentrations (0.01 mg/mL, 0.05 mg/mL, and 0.1 mg/mL) and control. At 0.15 mg/mL of iron-oxide nanoparticles, the highest inhibition (29 mm) was observed in* S. aureus* compared to* E. coli* and* P. aeruginosa*. The antibacterial activity of iron-oxide nanoparticles could be due to several mechanisms. The main mechanism suggested is the oxidative stress generated by ROS [26]. ROS includes superoxide radicals, hydroxyl radicals, hydrogen peroxide, and singlet oxygen, which may cause chemical damage to proteins and DNA in bacteria [27]. Secondly, electrostatic interactions between nanoparticles and bacterial cell membranes or cell membrane proteins can result in physical damage, which ultimately leads to bacterial cell death [26]. Other studies demonstrated that the small size of nanoparticles could contribute to their antibacterial effects [28, 29].

## 5. Conclusions

This study demonstrates that wettability of a biomaterial surface influences bacterial adhesion and biofilm growth. Polymer brush coated surfaces showed reduced bacterial adhesion compared to bare surfaces. A significant reduction in biofilm growth was observed due to the influence of iron-oxide nanoparticles on biofilms formed on polymer brush coated biomaterial surfaces. Thus combinational strategies such as polymer brush coating to biomaterial surface and influence of iron-oxide nanoparticles could significantly reduce biomaterial-associated infections.

## Figures and Tables

**Table 1:** Antibacterial activity of iron-oxide nanoparticles.

Microorganisms	Zone of inhibition (mm)			
	Concentration of iron-oxide nanoparticles (mg/mL)			
	0.01	0.05	0.1	0.15
*E*. *coli *	10	12	17	26
*P*. *aeruginosa *	11	13	16	28
*S*. *aureus *	13	16	19	29
